# Unoprostone reduces oxidative stress- and light-induced retinal cell death, and phagocytotic dysfunction, by activating BK channels

**Published:** 2011-12-30

**Authors:** Kazuhiro Tsuruma, Yuka Tanaka, Masamitsu Shimazawa, Yukihiko Mashima, Hideaki Hara

**Affiliations:** 1 Molecular Pharmacology, Department of Biofunctional Evaluation, Gifu Pharmaceutical University, Gifu, Japan; 2 Department of Ophthalmology, Keio University School of Medicine, 35 Shinano-machi, Shinjuku-ku, Tokyo, Japan

## Abstract

**Purpose:**

Unoprostone isopropyl (unoprostone) is a docosanoid currently used as an antiglaucoma agent. Unoprostone is known to have neuroprotective effects and to activate large conductance Ca^2+^-activated K^+^ (BK) channels. Recently, unoprostone has been tested in clinical studies as a therapeutic agent for retinitis pigmentosa (RP) and studies have demonstrated an improvement in retinal sensitivity and in the protection of central retinal sensitivity with its use. However, the mechanism of action underlying unoprostone’s protective effect in RP is not fully known. It is well known that the pathogenesis of RP can be accelerated by oxidative stress or light irradiation. Therefore, the current study investigated the effects and the underlying mechanism of action of unoprostone on oxidative stress- and light irradiation-induced damage in photoreceptor and retinal pigment epithelial cultures.

**Methods:**

The study used the mouse retinal cone-cell line 661W to investigate the effects of unoprostone and its major metabolite, unoprostone-free acid (M1), on oxidative stress- or light irradiation-induced cell death, and a human retinal pigment epithelial cell line (ARPE-19), was used to investigate the effects on light-induced disruption of phagocytotic function in a latex bead assay. Additionally, we examined whether the effects of unoprostone and M1 were mediated by BK channels using iberiotoxin, a selective inhibitor of BK channels.

**Results:**

Unoprostone and M1 protected against light- or H_2_O_2_-induced cell death in 661W cells, and against light-induced phagocytotic dysfunction in ARPE-19 cells. Additionally, iberiotoxin inhibited the protective effects of unoprostone and M1.

**Conclusions:**

These findings indicate that unoprostone has protective effects on oxidative stress- and light irradiation-induced damage in vitro and that these effects are mediated by activation of BK channels. This confirms that unoprostone represents a promising therapeutic agent for the treatment of RP and other retinal diseases.

## Introduction

Retinitis pigmentosa (RP) defines a set of hereditary retinal diseases that are characterized by the progressive degeneration of photoreceptors. RP is one of the major causes of visual handicaps or blindness, with the worldwide prevalence of RP being about 1 in 5,000 [1]. This represents more than 1 million affected individuals. RP patients typically lose night vision in adolescence, peripheral vision in young adulthood, and central vision in later life due to progressive photoreceptor degeneration. This photoreceptor degeneration starts with the loss of rods, generally preceding the loss of cones. Although some RP patients are treated with vitamins and antioxidants, including vitamin A [2] or docosahexanoic acid (DHA) [3], other therapeutic methods, such as photoreceptor-protective drugs, have been required in addition to treatment with those supplements.

Unoprostone is a synthetic docosanoid that has been shown to activate large conductance Ca^2+^-activated K^+^ (BK) channels and ClC-2 type chloride channels [4,5], but it has no significant prostaglandin receptor affinity [6,7]. Unoprostone (Rescula eyedrops; R-Tech Ueno, Tokyo, Japan) reduces intraocular pressure, and it is currently being used topically in patients with glaucoma or ocular hypertension. It has been shown that unoprostone lowers intraocular pressure in ocular-hypertensive patients by increasing aqueous outflow through the trabecular meshwork [8]. Endothelin-1 (ET-1) induces contraction of trabecular meshwork cells via an increase in intracellular calcium [Ca^2+^]_i_, and unoprostone induces a membrane hyperpolarization in trabecular meshwork cells via BK channel activation [9]. This counteracts the activation of voltage-gated calcium channels and calcium-triggered calcium release from intracellular stores, and thus, blocks the intracellular effects caused by ET-1 [5]. Unoprostone has been reported to alter the expression of matrix metalloproteinases (MMPs) [10], which are associated with intraocular pressure, cell death [11], and phagocytosis [12,13] in various tissues and cells. The neuroprotective effects of unoprostone have been examined in human neuronal cortical cells, a model system for studies of BK channel, activator-based neuroprotective agents [5]. In rat in vivo models, unoprostone has been shown to protect photoreceptors against constant light-induced damage [14]. Because Rescula eyedrops were reported to be effective for improving some functions of RP patients in Japan [15–17], unoprostone is being studied as a potential therapeutic agent for RP [18].

Photoreceptors are comprised of two types: rods that govern vision in low-light settings and cones that collect photons in ambient light and discern color differences. Typically in RP, rods degenerate first, followed by gradual cone-cell death. The etiology underlying most forms of RP are mutations associated with the photopigment metabolism. One of the most frequently occurring mutations affects the gene coding for the protein component of rhodopsin [19]. Some genes affected by RP are expressed not only in photoreceptors, but also in the retinal pigment epithelium (RPE) [20] and in tissues outside the eye [21,22]. To prevent the toxic effects of accumulated photo-oxidative products, photoreceptors undergo a daily renewal process wherein about 10% of their volume is shed and subsequently phagocytozed by adjacent RPE cells. It has been shown that the mutation of a receptor tyrosine kinase gene, which is found in RP patients, results in phagocytotic dysfunction in RPE cells and subsequent retinal degeneration [20].

It is also well known that the pathogenesis of RP is aggravated by oxidative stress [23,24] and light irradiation [25,26]. In particular, the retina consumes significant amounts of oxygen and produces a large amount of reactive oxygen species (ROS). In RP, photoreceptor apoptosis is the final common pathway leading to vision loss, and previous studies have reported that ROS induces photoreceptor apoptosis [27]. Similarly, studies have shown that light irradiation initiates photoreceptor apoptosis [27,28].

In this study, to reveal the mechanism of action underlying the protection of photoreceptor function by unoprostone in RP patients, we investigated the effects of unoprostone on oxidative stress- or light irradiation-induced cell damage using cone photoreceptor and RPE cell lines in vitro.

## Methods

### Materials

Unoprostone, the metabolite unoprostone-free acid (M1), latanoprost, and prostaglandin F_2α_ (PGF_2α_) were provided by R-tech Ueno. Amine-modified polystyrene fluorescent orange (1.0 μm latex beads, 4.98×10^10^ beads/ml) and iberiotoxin were obtained from Sigma-Aldrich (St. Louis, MO). H_2_O_2_ and phosphate buffer solution (PBS; 134 mM NaCl, 2.7 mM KCl, 8.1 mM Na_2_HPO_4_, and 1.47 mM KH_2_PO_4_; pH 7.4) were obtained from Wako (Osaka, Japan). Trolox, a vitamin E analog known as an antioxidant agent, was obtained from Cosmo Bio (Tokyo, Japan). Penicillin and streptomycin were obtained from Meiji Seika (Tokyo, Japan). Hoechst 33342 and propidium iodide (PI) were obtained from Daiichi Pure Chemicals (Tokyo, Japan).

### Cell culture

The mouse retinal cone-cell line 661W, a transformed mouse cone-cell line derived from mouse retinal tumors, was provided by Dr. Muayyad R. Al-Ubaidi (University of Oklahoma Health Sciences Center, Oklahoma City, OK). The cells were maintained in Dulbecco’s Modified Eagle’s Medium (DMEM; Sigma-Aldrich) containing 10% fetal bovine serum (FBS), 100 U/ml penicillin, and 100 μg/ml streptomycin. Cultures were maintained at 37 °C in a humidified atmosphere of 95% air and 5% CO_2_. The 661W cells were passaged by trypsinization every three to four days.

The human retinal pigment epithelial cell line (ARPE-19), a transformed human retinal pigment epithelial cell line, was obtained from American Type Culture Collection (Manassas, VA). The cells were maintained in DMEM/F-12 (Wako) containing 10% FBS, 100 U/ml penicillin, and 100 μg/ml streptomycin. Cultures were maintained at 37 °C in a humidified atmosphere of 95% air and 5% CO_2_. The ARPE-19 cells were passaged by trypsinization every three to four days. In this study, the ARPE-19 cells were used in an undifferentiated state.

### H_2_O_2_-induced cell death

The 661W cells were seeded at 2×10^3^ cells per well in 96-well plates and then incubated for 24 h. The entire medium was then replaced with fresh medium containing 1% FBS, and unoprostone, M1, latanoprost, and PGF_2α_ or trolox. Cultures were pretreated for 1 h, before H_2_O_2_ was added (at a final concentration of 0.3 mM). Unoprostone, M1, latanoprost, and PGF_2α_ were dissolved in dimethyl sulfoxide (DMSO; 10 mM stock solution) and diluted with PBS containing 1% DMSO (final concentration, 0.1%). Trolox, an antioxidant agent, was used as a positive (cell-protective) control in the experiments. Nuclear staining assays were performed after an additional 24 h of incubation.

To investigate whether the observed effects were mediated by BK channels, iberiotoxin, at a final concentration of 1 μM, was added with unoprostone or M1.

### Exposure of mouse retinal cone-cell line 661W cells to white light

The 661W cells were seeded at 2×10^3^ cells per well in 96-well plates and then incubated for 24 h. The entire medium was then replaced with fresh medium containing 1% FBS. Unoprostone, M1, latanoprost, and PGF_2α_ or trolox were added, and 1 h following treatment, the cells were exposed to 2,500 lx of white fluorescent light (C-FPS115D; Nikon, Tokyo, Japan) for 24 h at 37 °C. The luminance was measured using light meter LM-332 (As One, Osaka, Japan). Nuclear staining assays were performed after an additional 24 h of incubation. To investigate whether the observed effects were mediated by BK channels, iberiotoxin, at a final concentration of 1 μM, was added with unoprostone or M1.

### Nuclear staining assays

At the end of the culture period, Hoechst 33342 (λ_ex_=360 nm, λ_em_>490 nm) and PI (λ_ex_=535 nm, λ_em_>617 nm) were added to the culture medium for 15 min at final concentrations of 8.1 μM and 1.5 μM, respectively. Hoechst 33342 freely enters living cells and stains the nuclei of viable cells, as well as those that have suffered apoptosis or necrosis. Propidium iodide is a membrane-impermeable dye that is generally excluded from viable cells [29]. Images were collected using an Olympus IX70 inverted epifluorescence microscope (Olympus, Tokyo, Japan). We counted the total number of cells and calculated the percent of PI-positive cells as a measure of dead cells.

### Mitochondrial membrane potential

After 24 h, the mitochondrial membrane potential was measured in light-exposed 661W cells using the JC-1 Mitochondrial Membrane Potential Assay Kit (Cayman Chemical Company, Ann Arbor, MI) according to the manufacturer’s protocol. Images were collected using a fluorescence microscope (Keyence, Osaka, Japan), which detects healthy cells with mainly JC-1 J-aggregates (excitation/emission=540/605 nm) and unhealthy or apoptotic cells with mainly JC-1 monomers (excitation/emission=480/510 nm). Merged cells were determined to be pre-apoptotic (early or middle state of transition to cell death) cells.

### Phagocytosis assays

ARPE-19 cells were seeded at 1×10^5^ cells per well in 24-well plates and then incubated for 48 h. The cells reached confluence, which was confirmed with a microscope (Olympus), and the entire medium was then replaced with fresh medium containing 1% FBS. Unoprostone, M1, latanoprost, and PGF_2α_ or trolox were added, and 1 h later, the cells were exposed to 2,500 lux of white fluorescent light (Nikon) for 48 h at 37 °C. After 48 h of incubation, 1.4 μl latex beads diluted with 450 μl medium were added to 50 μl/wells (at a final concentration of 1×10^6^ beads/wells) and incubated for 4 h. Subsequently, the cells were washed five times with PBS to remove extracellular latex beads and exposed to 4% paraformaldehyde (PFA; Wako) for 10 min. After 10 min, the cells were washed again with PBS to remove PFA, and Hoechst 33342 was added to the culture medium for 15 min, at final concentrations of 8.1 μM, for nuclear staining. Images were collected using a fluorescence microscope (Keyence). We counted the total number of cells and the number of intracellular latex beads and calculated the percentage of intracellular latex beads relative to the total number of cells. To investigate whether the observed effects were mediated by BK channels, iberiotoxin, at a final concentration of 1 μM, was added with unoprostone or M1.

### Statistical analyses

Data are presented as means±standard error of the mean (SEM). Statistical comparisons were made using a one-way ANOVA (ANOVA) followed by a Tukey’s test, a Dunnett’s test, or a Student’s *t*-test. A value of p<0.05 was considered to indicate statistical significance.

## Results

### Unoprostone and M1 suppressed H_2_O_2_-induced photoreceptor cell death

**Figure 1:**
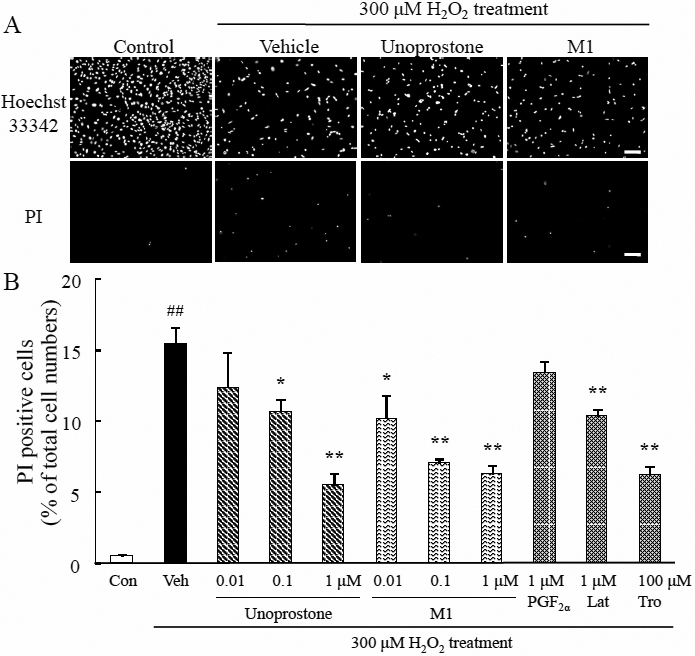
Unoprostone and M1 suppressed H_2_O_2_-induced photoreceptor cell death. **A**: Representative fluorescence microscopic images showing nuclear staining for Hoechst 33342 and PI after 24 h H_2_O_2_ treatment. Unoprostone (0.01–1 µM), M1 (0.01–1 µM), PGF_2α_ (1 µM), and latanoprost (1 µM) or trolox (100 µM) were added 1 h before H_2_O_2_ treatment. **B**: The number of cells exhibiting PI fluorescence was counted, and expressed as the percentage of PI positive cells to Hoechst 33342 positive cells. Data are expressed as mean±SEM (n=6). * p<0.05, ** p<0.01 versus vehicle; ^##^ p<0.01 versus control (Dunnett’s test). Con: control; Veh: vehicle; Lat: latanoprost; Tro: trolox. Scale bar represents 100 µm.

We examined the effect of unoprostone and M1 on H_2_O_2_-induced photoreceptor cell death. Representative photographs of Hoechst 33342 and PI staining are shown in Figure 1A. Hoechst 33342 stains all cells (live and dead cells), whereas PI stains only dead cells. Pretreatment with unoprostone at concentrations of 0.01 to 1 μM protected against H_2_O_2_-induced cell death in a concentration-dependent manner; the effect was significant at 0.1 μM (p<0.05) and 1 μM (p<0.01; n=6) concentrations. Pretreatment with M1 at concentrations of 0.01 to 1 μM also protected against H_2_O_2_-induced cell death in a concentration-dependent manner; the effect was significant at concentrations of 0.01 μM (p<0.05), 0.1 μM, and 1 μM (p<0.01; n=6). Latanoprost at 1 μM reduced cell death (p<0.01; n=6). Trolox at a concentration of 100 μM inhibited cell death (p<0.01; n=6; Figure 1B).

### Unoprostone and M1 reduced cell death induced by white light in mouse retinal cone-cell line 661W cells

**Figure 2:**
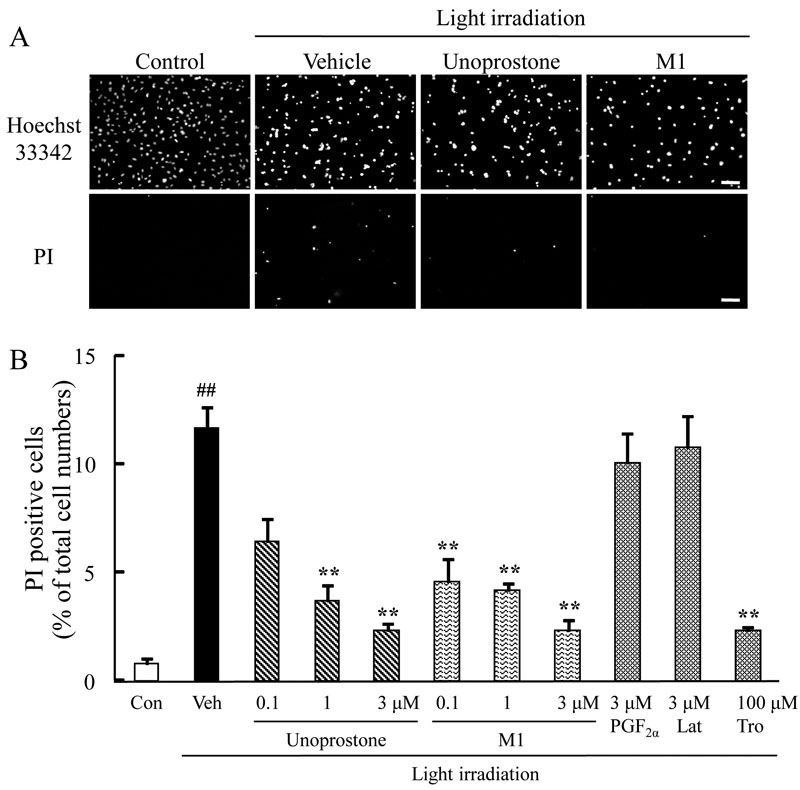
Unoprostone and M1 reduced cell death induced by white light in mouse retinal cone-cell line 661W cells. **A**: Representative fluorescence microscopic images show nuclear staining for Hoechst 33342 and PI after 24 h light irradiation. Unoprostone (0.1–3 µM), M1 (0.1–3 µM), PGF_2α_ (3 µM), latanoprost (3 µM) or trolox (100 µM) were added before light irradiation. **B**: The number of cells exhibiting PI fluorescence was counted, and positive cells were expressed as the percentage of PI to Hoechst 33342. Data are expressed as mean±SEM (n=6). ** p<0.01 versus vehicle; ^##^ p<0.01 versus control (Dunnett’s test). Con: control; Veh: vehicle; Lat: latanoprost; Tro: trolox. Scale bar represents 100 µm.

**Figure 3:**
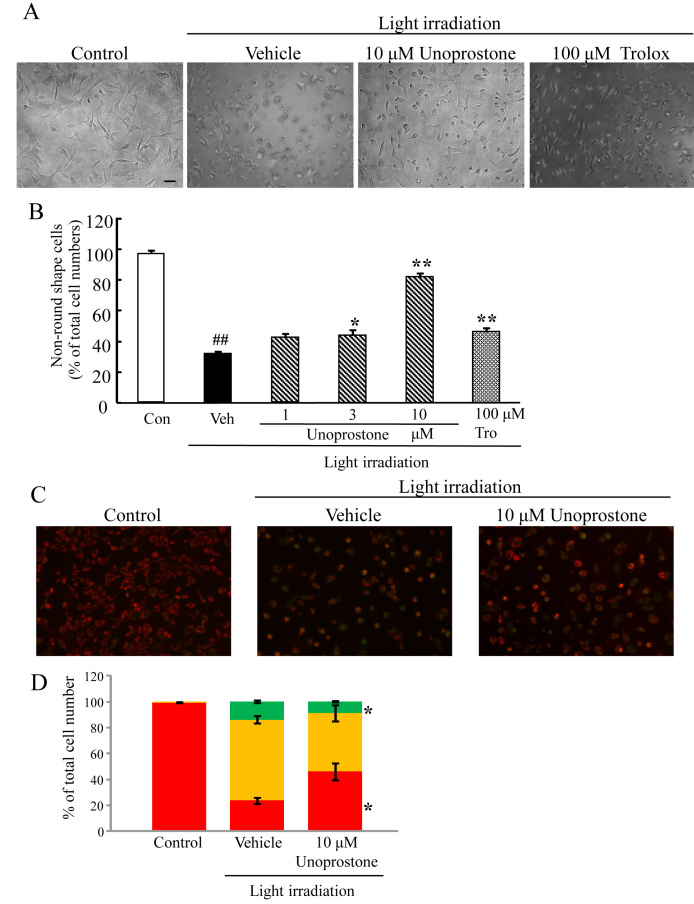
Unoprostone reduced morphological change induced by light irradiation in mouse retinal cone-cell line 661W cells. **A**: Representative images show cell morphologies. Control cells had an elongated appearance with extended processes, whereas cell morphology was altered to a round shape after 24 h light irradiation. Cells were treated with 10 µM of unoprostone and 100 µM of trolox before light irradiation. **B**: Unoprostone (1–10 µM) was added before light irradiation. The number of nonround-shaped cells was counted, and expressed as the percentage of total cell numbers. **C**: Representative images show JC-1 stained cells. Healthy cells with mainly JC-1 J-aggregates can be detected as red cells, and unhealthy or apoptotic cells with mainly JC-1 monomers can be detected as green cells. Merged (orange) cells were determined to be pre-apoptotic (early or middle state of transition to cell death) cells. **D**: The number of cells with each color were counted and expressed as the percentage of total cell numbers (red bar=red fluorescence cells, orange bar=merged cells, and green bar=green fluorescence cells, respectively). Data are expressed as mean±SEM (**B**; n=6, **D**; n=3). * p<0.05, ** p<0.01 versus vehicle; ## p<0.01 versus control (Dunnett’s test). Con: control; Veh: vehicle; Tro: trolox. Scale bar represents 100 µm.

We examined the effects of unoprostone and M1 on white light-induced cell death. White light irradiation can lead to 661W cell death [30,31]. Representative photographs of Hoechst 33342 and PI staining are shown in Figure 2A. Pretreatment with unoprostone at concentrations of 0.1 to 3 μM protected against light-induced cell death in a concentration-dependent manner; the effect was significant at the 1 and 3 μM concentrations (p<0.01; n=6). Pretreatment with M1 at concentrations of 0.1 to 3 μM also protected against light-induced cell death in a concentration-dependent manner; the effect was significant at the 0.1, 1, and 3 μM concentrations (p<0.01; n=6). In contrast, latanoprost or PGF_2α_ at a concentration of 3 μM did not affect cell death (p=0.64 and 0.36, respectively; n=6). Trolox at a concentration of 100 μM inhibited cell death (p<0.01; n=6; Figure 2B). Moreover, unoprostone reduced the morphological change—the transition to a rounder shape—that is potentially indicative of a pre-apoptotic stage (Figure 3A,B), and it significantly inhibited the low mitochondrial membrane potential and cell death (Figure 3C,D) induced by light irradiation.

### Unoprostone and M1 suppressed phagocytotic dysfunction induced by white light in ARPE-19 cells

**Figure 4:**
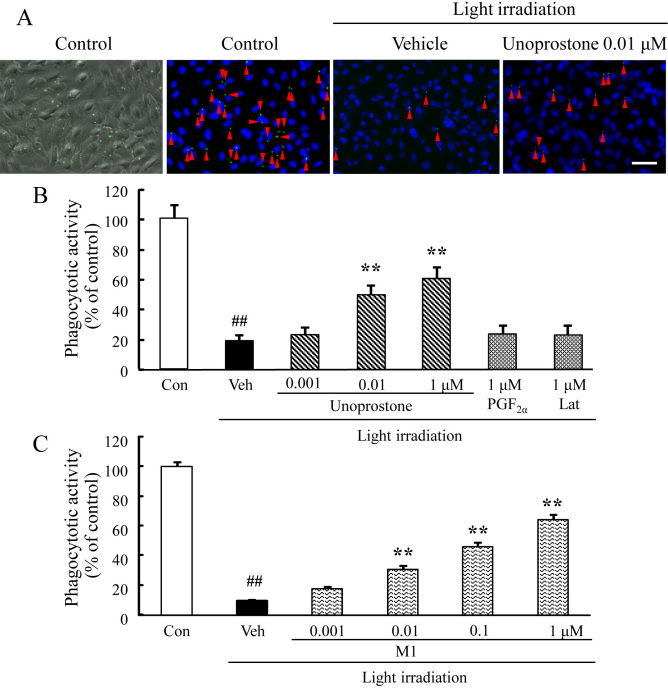
Unoprostone and M1 suppressed phagocytotic dysfunction induced by white light in ARPE-19. **A**: Representative fluorescence microscopy shows latex beads and morphology, or latex beads and nuclear staining for Hoechst 33342 after 48 h light irradiation. Arrowheads indicate the fluorescent beads. **B**, **C**: Unoprostone (0.001–1 µM), M1 (0.001–1 µM), and PGF_2α_ (1 µM) or latanoprost (1 µM) were added before light irradiation. Latex beads were added after light irradiation, and 4 h later, they were removed by washing. The number of intracellular latex beads was counted, and phagocytotic activity was expressed as the percentage of latex beads to total cell numbers. Data are expressed as mean±SEM (PGF_2α_ and Lat; n=4, unoprostone and M1; n=8). ** p<0.01 versus vehicle; ^##^ p<0.01 versus control (Dunnett’s test). Con: control; Veh: vehicle; Lat: latanoprost. Scale bar represents 50 µm.

To investigate the effect of unoprostone on phagocytotic activity, a crucial function of RPE, ARPE-19 cells were exposed to white light irradiation, which can lead to the phagocytotic dysfunction of RPE cells [32]. Representative fluorescence microscopy showing intracellular latex beads and morphology, or latex beads and nuclear staining for Hoechst 33342, are shown in Figure 4A. Pretreatment with unoprostone at concentrations of 0.001 to 1 μM suppressed light-induced phagocytotic dysfunction in a concentration-dependent manner; the effect was significant at the 0.01 and 1 μM concentrations (p<0.01; n=8). In contrast, latanoprost and PGF_2α_ at a concentration of 1 μM did not affect phagocytotic dysfunction (p=0.61 and 0.57, respectively; n=4; Figure 4B). Pretreatment with M1 at concentrations of 0.001 to 1 μM also suppressed light-induced phagocytotic dysfunction in a concentration-dependent manner; the effect was significant at the 0.01, 0.1, and 1 μM concentrations (p<0.01; n=8; Figure 4C). On the other hand, trolox at a concentration of 100 μM did not affect phagocytotic dysfunction (data not shown).

### Iberiotoxin attenuated the protective effects of unoprostone and M1 against mouse retinal cone-cell line 661W cell death

**Figure 5:**
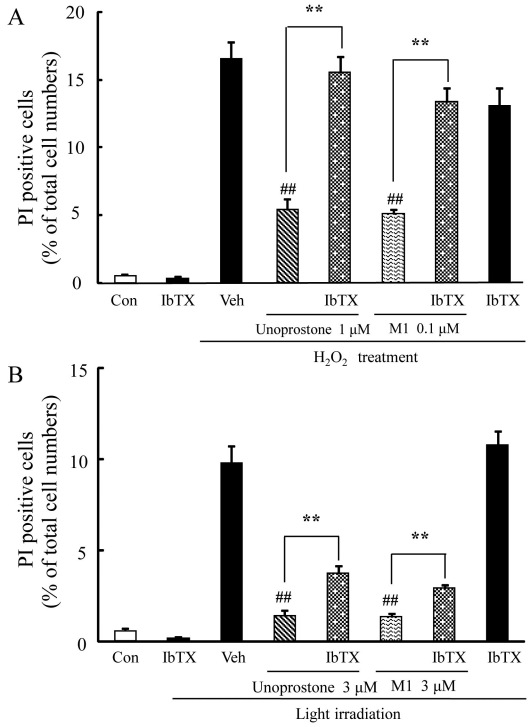
Iberiotoxin attenuated protective effects of unoprostone and M1 on mouse retinal cone-cell line 661W cell death. **A**: Unoprostone (1 µM), and M1 (0.1 µM) or iberiotoxin (1 µM) were added 1 h before H_2_O_2_ treatment. **B**: Unoprostone (3 µM), M1 (3 µM) or iberiotoxin (1 µM) were added just before light irradiation. The number of cells exhibiting PI fluorescence was counted, and positive cells were expressed as the percentage of PI to Hoechst 33342. Data are expressed as mean±SEM (n=6). ** p<0.01 versus Unoprostone or M1 treatment; ^##^ p<0.01 versus vehicle (Student’s *t*-test). Con: control; Veh: vehicle; IbTX: iberiotoxin.

Iberiotoxin, an inhibitor of BK channels, was used with unoprostone or M1 to investigate the relation between BK channels and cell death in 661W cells. The protective effects of unoprostone and M1 on H_2_O_2_-induced cell death were significantly attenuated by iberiotoxin (p<0.01; n=6; Figure 5A). However, iberiotoxin by itself did not affect H_2_O_2_-induced cell death (p=0.20; n=6). Similarly, the protective effects of unoprostone and M1 on light-induced cell death were significantly attenuated by iberiotoxin (p<0.01; n=6; Figure 5B), whereas iberiotoxin by itself did not affect light-induced cell death (p=0.43; n=6).

### Iberiotoxin attenuated phagocytotic activity recovered by unoprostone and M1

**Figure 6:**
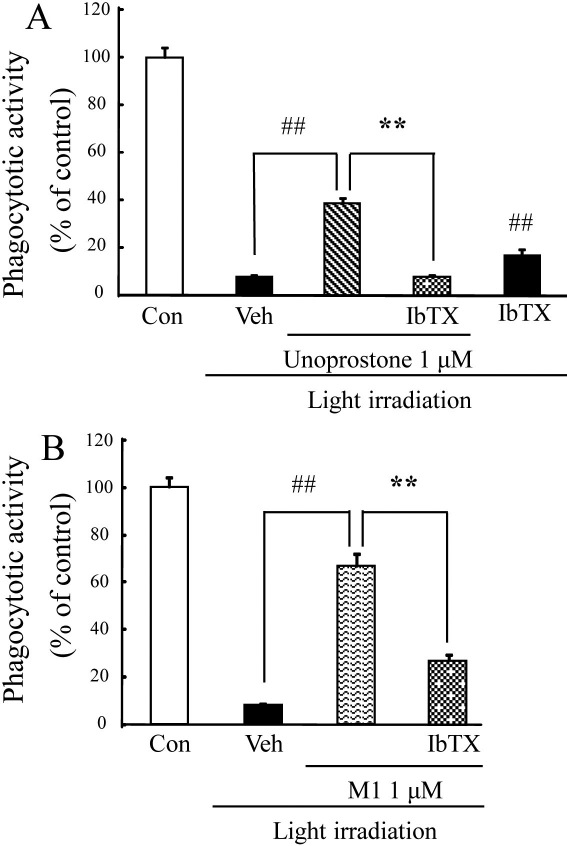
Iberiotoxin attenuated phagocytotic activity recovered by unoprostone and M1. **A**, **B**: Unoprostone (1 µM), and M1 (1 µM) or iberiotoxin (1 µM) were added just before light irradiation. Latex beads were added just after light irradiation, and 4 h later, they were removed by washing. The number of latex beads was counted, and phagocytosis was expressed as the percentage of latex beads to total cell numbers. Data are expressed as mean±SEM (n=8). ** p<0.01 versus Unoprostone or M1 treatment; ^##^ p<0.01 versus vehicle (Student’s *t*-test). Con: control; Veh: vehicle; IbTX: iberiotoxin.

Iberiotoxin was also used to investigate the relation between BK channels and light-induced phagocytotic dysfunction in ARPE-19 cells. The protective effects of unoprostone and M1 on light-induced phagocytotic dysfunction were significantly attenuated by iberiotoxin (p<0.01; n=8), although iberiotoxin by itself inhibited light-induced phagocytotic dysfunction (p<0.01; n=8; Figure 6).

## Discussion

First, using 661W cells, we investigated the effect of unoprostone on oxidative stress, which is known to accelerate the progression of RP [23,24]. The results showed that unoprostone has a protective effect against H_2_O_2_-induced cell death in this cell line. In RP, a large number of mutations result in rod-cell death, followed by the gradual death of cone cells [33]. Cone-cell degeneration is responsible for the gradual constriction of the central visual field and eventual blindness; therefore, visual acuity and the quality of life of late stage RP patients crucially depend on the rate of cone-cell degeneration. It has been proposed that the death of rod cells results in decreased oxygen consumption and hyperoxia in the outer retina, resulting in gradual cone-cell death [34]. This hypothesis suggests that protection of cone cells from oxidative stress is necessary to improve the visual quality of life of RP patients. Therefore, this study investigated whether unoprostone produced antioxidant activities. However, unoprostone did not produce radical scavenging activity in H_2_O_2_ or hydroxyl radical (data not shown).

Unoprostone protected photoreceptor cells against constant light-induced damage in a rat model [14]. In the present study, unoprostone also prevented the 661W cell death induced by light irradiation. These results suggest that unoprostone directly affects photoreceptors, promoting their survival under conditions of oxidative- or light-induced metabolic stress. Moreover, we observed that white light could induce a morphological change in 661W cells, making the cell shape round in appearance, thus to resemble apoptotic cells, but without inducing cell death. Unoprostone suppressed this alteration (Figure 3A,B), further indicating that unoprostone prevents light-exposed cells from entering the apoptotic pathway. Interestingly, this effect was unique to unoprostone and not seen with the radical scavenger trolox, although the result of trolox at a concentration of 1 mM was equal to that of trolox at a concentration of 100 µM in terms of light-induced cell death and morphological change (data not shown). Unoprostone might not only protect against apoptotic cell death under conditions of oxidative stress, but also affect the function of photoreceptors by a mechanism other than antioxidant activity. On the other hand, it has been reported that a BK channel opening agent directly affects mitochondria [35]. Considering the previous report and our JC-1 study (Figure 3C,D), unoprostone may affect BK channels existing in mitochondria, and the protective effect of unoprostone on light-induced morphological change may be exerted by the reduction of mitochondrial damage.

Next, a cultured RPE cell line was used to investigate the effect of unoprostone on light-induced phagocytotic dysfunction. This study used latex beads to investigate the phagocytosis in RPE cells without photoreceptor outer segments (POS). Although POS is important for retinal phagocytosis, POS-independent phagocytosis has been reported [36–38], and we measured the phagocytosis by reference to these reports. RPE cells are at risk of oxidative injury due to the high level of light exposure and the generation of ROS by the photoreceptor outer segments [39]. Previous studies reported that light irradiation induced oxidative stress in RPE cells [40,41], and that activation of α2 AMP-activated protein kinase contributed to the inhibition of RPE cell phagocytosis by oxidative stress [42,43]. However, the results of the current study have shown that trolox did not ameliorate phagocytotic dysfunction; therefore, oxidative stress may not contribute to light-induced phagocytotic dysfunction. Phagocytosis in RPE is known to depend on diurnal rhythm [44]. Previously, light exposure was found to reduce phagocytosis in vitro, whereas it recovered in the dark [45]. Thus, the protective effect of unoprostone against phagocytotic dysfunction may not be related to the antioxidant effect but to different aspects of its mechanism of action, especially under light irradiation. Further experiments will be needed to elucidate the mechanism of action of unoprostone-regulated molecules such as proto-oncogene tyrosine-protein kinase MER, known as an RP-related gene [20], in phagocytosis [46].

Following ocular instillation, unoprostone is immediately metabolized, during corneal passage, to M1 [47]. M1 is the major intraocular metabolite of unoprostone, and responsible for its pharmacological effects on target tissues in vivo. The current results reveal that M1 is responsible not only for unoprostone’s intraocular pressure-lowering effect but also for its neuroprotective activity, since the protective effects of M1 are equal to or higher than those of unoprostone.

In 1977, it was reported that intraocular pressure was reduced by prostaglandins applied topically to the eyes of conscious rabbits [48]. Furthermore, intraocular pressure is reduced by increasing aqueous humor outflow via PGF_2α_-receptor (FP receptor) activation [49]. In previous reports, it has been suggested that unoprostone has a distinctly different mechanism of action from latanoprost and PGF_2α_ as FP-receptor agonists [5]. The present study revealed that unoprostone also has different effects from latanoprost in regard to light- or oxidative stress-induced retinal damage. This study’s results regarding the effects of latanoprost and PGF_2α_ are in line with those reports, although the mechanism underlying the moderate protective effect of latanoprost against oxidative stress-induced retinal cell death remains unclear. On the other hand, latanoprost has been reported to have a protective effect on glutamate- or hypoxia-induced retinal ganglion cell death, whereas unoproston did not have a protective effect [50]. Considering these reports and the current data, the protective effects of unoprostone may be cell- and stimulus-specific.

It is well known that unoprostone’s mechanism of action involves activation of BK channels [9]. Previous studies have indicated that resveratrol directly stimulated BK channel activity in vascular endothelial cells [51], and significantly reduced oxidative stress-induced inhibition of phagocytosis in human RPE cells, which was also linked to activation of BK channels [38]. The EC_50_ for activation of BK channels by unoprostone was approximately 0.6 nM in cortical and retinal cultured neurons [4,5]. In contrast, it was reported that the EC_50_ for FP-receptor binding was 5.9 μM [7], and for Ca^2+^ mobilization was approximately 1 μM [52], indicating a very low affinity of unoprostone to prostaglandin receptors. Taken together, the findings in the present study suggest that the protective effect of unoprostone on light-induced phagocytotic dysfunction is mediated by BK channel activation, since the effect of unoprostone was observed at low concentrations and attenuated by iberiotoxin. On the other hand, unoprostone has been reported to alter the expression of MMPs and their inhibitors, and this expression pattern is different from that of latanoprost or bimatoprost [10]. Not only MMPs but also other protein expression and/or other mechanisms, such as the inhibition of calcium influx and the protection of the mitochondrial membrane potential, may be involved in unoprostone’s protective effects against light-induced cell death and morphological changes.

In conclusion, our results suggest that unoprostone protects photoreceptors and RPE cells against oxidative- and light-induced damage by a mechanism different from antioxidant activity. The different mechanism of action suggests that future therapies may include a combined application of antioxidant agents and unoprostone to optimize treatment for RP. Unoprostone’s combined protective effects on photoreceptors and RPE cell function are also promising features for the treatment of RP.
